# Real-world data on the prevalence of BRCA1/2 and HRR gene mutations in patients with primary and metastatic castration resistant prostate cancer

**DOI:** 10.1007/s00345-024-05188-7

**Published:** 2024-08-22

**Authors:** Moritz Hommerding, Oliver Hommerding, Marit Bernhardt, Tobias Kreft, Christine Sanders, Verena Tischler, Patrick Basitta, Natalie Pelusi, Anna-Lena Wulf, Carsten-Henning Ohlmann, Jörg Ellinger, Manuel Ritter, Glen Kristiansen

**Affiliations:** 1 Institute of Pathology, University Hospital Bonn (UKB), Venusberg-Campus 1, Bonn, 53127 Germany; 2 Department of Urology, University Hospital Bonn, Bonn, Germany; 3 Department of Urology, Johanniter-Kliniken Bonn, Bonn, Germany

**Keywords:** Prostate cancer, PARPi, BRCA2, HRR, Olaparib

## Abstract

**Purpose:**

This study seeks to contribute real-world data on the prevalence of BRCA1/2 and HRR gene mutations in prostate cancer.

**Methods:**

We compiled sequencing data of 197 cases of primary and metastatic prostate cancer, in which HRR mutation analysis was performed upon clinical request within the last 5 years. All cases were analyzed using a targeted NGS BRCAness multigene panel, including 8 HRR genes (ATM, BRCA1, BRCA2, CDK12, CHEK2, FANCA, HDAC2, PALB2).

**Results:**

Our findings reveal a prevalence of potentially targetable mutations based on FDA criteria of 20.8%, which is comparable to the literature. However, the frequency of targetable BRCA2 mutations within our cohort was lower than reported for mCRPC and ATM and CHEK2 mutations were more prevalent instead. Thus, while 20.8% (*n* = 38) of the cases meet the criteria for olaparib treatment per FDA approval, only 4.9% (*n* = 9) align with the eligibility criteria according to the EMA approval.

**Conclusion:**

This study offers valuable real-world insights into the landscape of BRCA1/2 and HRR gene mutations and the practical clinical management of HRR gene testing in prostate cancer, contributing to a better understanding of patient eligibility for PARPi treatment.

## Introduction

In May 2020, the Food and Drug Administration (FDA) granted approval for olaparib (Lynparza) for patients diagnosed with metastatic castration-resistant prostate cancer (mCRPC) possessing pathogenic or suspected pathogenic somatic or germline mutations in homologous recombination repair (HRR) genes. Specifically, this approval was extended to patients who had experienced disease progression following treatment with enzalutamide or abiraterone. Subsequently, the European Medicines Agency (EMA) endorsed olaparib for individuals harboring pathogenic or suspected pathogenic somatic or germline mutations in BRCA1/2 in November the same year. The phase 3 PROfound trial formed the basis for these regulatory decisions, demonstrated significant imaging-based progression-free survival (ibPFS) and overall survival benefits with olaparib in patients with mCRPC harboring BRCA1, BRCA2, and ATM mutations after disease progression on a next-generation hormonal agent [1] In addition, significant ibPFS and a trend towards prolonged overall survival was seen in patients harboring alterations in other HRR genes [1, 2].

Furthermore, the PROpel trial’s outcomes led to the approval of Olaparib in combination with abiraterone for mCRPC patients [3]. According to the EMA approval, BRCA1/2 and HRR gene testing is not mandated in this clinical context and chemotherapy must be contraindicated in these patients [4]. In contrast, proof of a BRCA1/2 mutation is required according to the FDA approval [5, 6]. In addition to the mutation test on tumor tissue, a mutation test on circulating tumor DNA (FoundationOne Liquid CDx; Foundation Medicine) was carried out in the PROpel trial, which allowed an increased detection of HRR gene alterations. [3]Other recent studies have led to approval of niraparib plus abiraterone and talazoparib plus enzalutamide in the same clinical setting [7, 8].

The prevalence of defects in DNA repair genes, particularly alterations in HRR genes, ranges from 19 to 33% among individuals with prostate cancer, depending on whether primary or metastatic tumor tissue is considered [9–11] BRCA2 alterations emerge as the most prevalent in metastatic disease [9–13].

An integrative assessment of 333 primary prostate cancers within the TCGA Research Network revealed inactivating alterations in DNA repair genes in approximately 19% of cases [11]. Notably, alterations in FANCD2 were most prevalent (7%), followed by ATM (4%), BRCA2 (3%), RAD51C (3%), CDK12 (2%), and BRCA1 (1%). While missense and truncating mutations were predominant for BRCA1, BRCA2, ATM, and CDK12, FANCD2 and RAD51C exhibited mostly hemizygous and homozygous deletions.

The largest cohort of patients with mCRPC has been investigated by Robinson et al. [9] Using whole exome and transcriptome sequencing, targetable HRR alterations were found in 19.3% of the patients. BRCA2 alterations were most prevalent (12.7%) and 20% presented as homozygous deletions.

The TOPARP-A trial reported HRR gene alterations in 33% of cases in their cohort, with BRCA2 alterations being most common (14.3%) [14]. ATM alterations were found in 10.2% of patients, with diverse mutation types observed. The PROfound trial reported mutations predominantly in BRCA2, followed by ATM, CDK12, and few in CHEK2 [1].

The prevalence of germline mutations varies from 4.6% in localized disease to 16% in metastatic disease [9, 15, 16]. An unselected cohort of 692 patients with mCRPC demonstrated that about 11.8% harbored germline mutations in DNA repair genes, predominantly in HRR genes [15]. Of these, BRCA2 mutations were the most prevalent (5.3% of 11.8%). The PROREPAIR-B study found germline DNA repair mutations in 16% of 419 patients, primarily in BRCA2 (21%), ATM (12%), and BRCA1 (6%) [16].

Post the FDA and EMA approval of olaparib, numerous laboratories have initiated mutation analysis services for BRCA1/2 and HRR-related genes. This study aims to contribute real-world data on the prevalence of BRCA1/2 and HRR gene mutations in prostate cancer.

## Materials and methods

### Case selection and clinicopathological data of the cohort

197 cases of primary and metastatic prostate cancer were analyzed using a targeted NGS BRCAness multigene panel between September 2018 and December 2023 at the Institute of Pathology, University Hospital Bonn, upon clinical request. The clinicopathological data of the cohort is summarized in Table 1.

**Table 1:** Clinicopathological data of the cohort

Clinical features	Finding (*n* = 197)
Age, median (range), years	71 (39–89)
Initial PSA, ng/ml, median (range)	30 (0.3–10.000)
Pathological parameters	
Grade Group at initial diagnosis, median (range)	4 (1–5)
Grade group 1, n (%)	3 (1.5%)
Grade group 2, n (%)	18 (9.1%)
Grade group 3, n (%)	12 (6.1%)
Grade group 4, n (%)	37 (18.8%)
Grade group 5, n (%)	97 (49.2%)
unknown, n (%)	29 (14.2%)
RPE parameters	Finding (*n* = 55, 27.9%)
Organ-confined disease, n (%)	12 (21.8%)
Extraprostatic extension, n (%)	13 (23.6%)
Seminal vesicle involvment, n (%)	29 (52.7%)
Infiltration of bladder, pelvic wall, rectum	1 (1.8%)
Lymph node positive, n (%)	21 (38.2%)
Positive margins, n (%)	28 (50.9%)
Tumor sample age, mean, years (range, sd)	2.1 (0–21, 3.7)
Time from initial diagnosis to HRR testing, years (range, sd)	5.4 (0–22, 5.2)

**Fig. 1:**
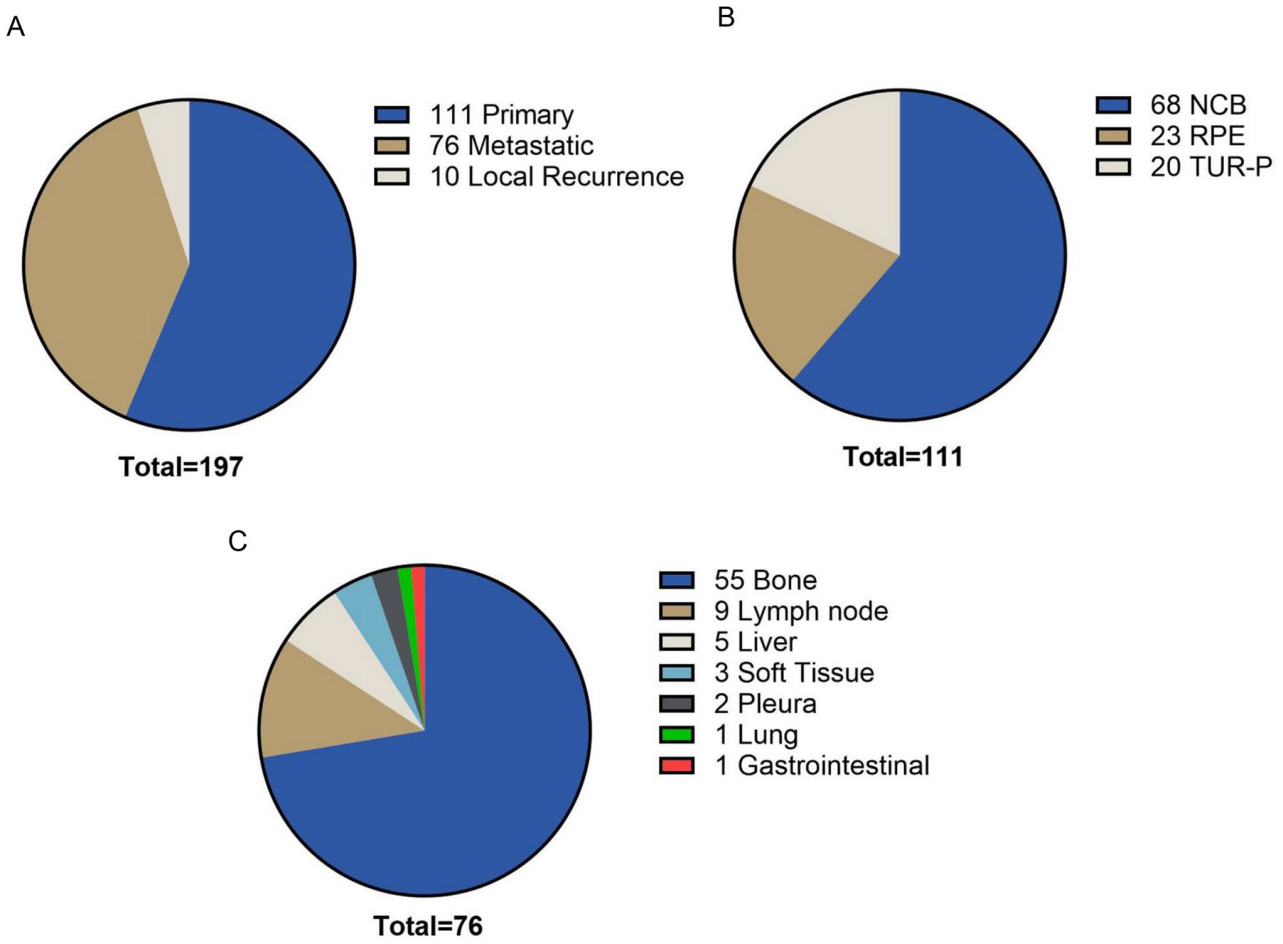
The distribution of tumor samples tested for HRR alterations. (**A**) Primary tumor samples were the preferred type of tumor tissue tested for HRR alterations (*n* = 111), followed by metastatic tumor tissue (*n* = 76) and tumor tissue from local recurrences (*n* = 10). (**B**) Needle core biopsies (NCB) from the initial diagnosis of prostate cancer were the preferred material tested for HRR alteration (*n* = 68), followed by radical prostatectomy specimen (RPE) (*n* = 23) and transurethral resection specimen (TUR-P) (*n* = 20). (**C**) Bone metastases were the most common material tested for HRR alterations (*n* = 55), followed by distant lymph node metastases (*n* = 9), liver metastases (*n* = 5), soft tissue metastases (*n* = 3), pleural metastases (*n* = 2). Lastly, one lung metastasis and one gastrointestinal metastasis were analyzed

The median patient’s age was 71 (range 39–89). The median initial PSA value was 30 ng/ml (range 0.3–10.000). The median ISUP grade at the initial diagnosis was 4 (range 1–5). The samples included primary tumor tissue, metastatic tumor tissue and tissue from local recurrences (Fig. 1). The majority of patients were clinically mCRPC at the timepoint of HRR testing (*n* = 171, 86.8%). For a subset of the patients a recent tumor sample obtained in the setting of mCRPC was analyzed (40.1% (*n* = 79)). For the remaining patients, primary tissue from the initial diagnosis was investigated. Pathological data from radical prostatectomy specimen was available for 55 patients. 42 (76.4%) patients showed non-organ-confined disease, 21 (38.2%) showed positive lymph nodes and 28 (50.9%) had positive resection margins. The mean tumor sample age was 2.1 years (range 0–21). The mean time from the initial diagnosis to molecular testing was 5.4 years (range 0–22).

All data was acquired prospectively during patient care, rendering a specific ethics votum not necessary.

### Sample preparation, DNA isolation, sequencing and data analysis

Suitable tumor areas were grossly dissected after histological review of the slides by a board certified pathologist and the epithelial cellularity was recorded. DNA extraction was conducted with the Maxwell RSC DNA FFPE Kit (Promega, Madison, Wisconsin, US). Next generation sequencing was performed using a QIAseqTM targeted DNA BRCAness custom panel (Qiagen, Hilden, Germany) including 8 genes involved in homologous recombination repair as follows: ATM, BRCA1, BRCA2, CDK12, CHEK2, FANCA, HDAC2, PALB2. All coding sequences were analyzed comprising a total of 38,450 bp. Generation of multiplex amplicons and library preparation including unique molecular identifiers was performed according to the manufacturer’s recommendations. Next generation sequencing was performed on a MiSeq sequencer with a coverage of > 100 (Illumina, San Diego, US). Data was analyzed with the CLC Genomics Workbench/Server (Qiagen Bioinformatics, Hilden, Germany). Coding-synonymous variants, intronic variants not involving consensus splice sites and variants with a population allele frequency of > 1% were filtered. Variants called benign in databases were not reported. Pathogenic and likely pathogenic mutations with an allele frequency of ≥ 5% were reported. Classification of variants was performed according to the following databases: dbSNP, ExAC, COSMIC, ClinVar, OncoKB, UMD-BRCA1/2 Databases (University of Utah BRCA1/2 databases).

### Statistical analysis and software

Statistical analysis was performed using Prism, Version 10.2.2 (GraphPad Software, Boston, US). Chi-square test was used for comparing the prevalence of mutations in metastatic and primary tumor tissue. Graphs were generated with Prism, Version 10.2.2 (GraphPad Software, Boston, US).

## Results

In this study, we compiled BRCA1/2 and HRR sequencing data of 197 cases of primary and metastatic prostate cancer.

The distribution of tumor tissue tested revealed a predominant analysis of primary tumor tissue (*n* = 111, 56.3%) in contrast to metastatic tumor tissue (*n* = 76, 38.6%) and tumor tissue from local recurrences (*n* = 10, 5.1%) (Fig. 1A). The preferred sources for primary tumor testing included prostate needle core biopsies (*n* = 68, 34.5%), followed by radical prostatectomy (RPE) specimens (*n* = 23, 11.7%) and transurethral resection (TUR) specimens (*n* = 20, 10.2%) (Fig. 1B).

Bone metastases (*n* = 55, 27.9%) were the most common metastatic tumor tissue followed by distant lymph node metastases (*n* = 9, 4.6%), hepatic metastases (*n* = 5, 2.5%) and infrequent metastatic sites such as pleura, lung, gastrointestinal tract or soft tissue metastases (in total *n* = 7, 3.6%) (Fig. 1C).

The majority of patients were clinically mCRPC at the timepoint of HRR testing (*n* = 171, 86.8%). However, only in a subset of those patients a recent tumor sample obtained in the setting of mCRPC was analyzed (40.1% (*n* = 79)). For the remaining patients, primary tissue from the initial diagnosis, such as needle core biopsies, RPE specimen and TUR specimen, was investigated, which is considered hormone-sensitive tumors.

A subset of cases (*n* = 14, 7.1%) exhibited insufficient DNA quality. Intriguingly, almost all (*n* = 13) instances of insufficient quality pertained to primary tumor samples. The mean sample age of the primary samples with insufficient DNA quality was greater compared to the mean sample age of the primary samples with sufficient DNA quality (8.1 years vs. 3.0 years). Therefore, the insufficient quality can likely be attributed to the sample age. In contrast, metastatic tissue samples uniformly exhibited adequate sequencing quality. One case of a local recurrence, sampled via needle core biopsy, showed insufficient quality as well.

Among the 183 cases subjected to comprehensive variant analysis, 79 cases (43.2%) manifested at least one suspected benign variant, variant of unknown significance, suspected pathogenic mutation or pathogenic mutation. Unambiguous benign variants were not reported. The distribution of cases with mutations indicated a prevalence of single mutations in 55 cases (30.0%), double mutations in 17 cases (9.3%), and triple mutations in 7 cases (3.8%), accounting for a total of 110 mutations.

**Fig. 2:**
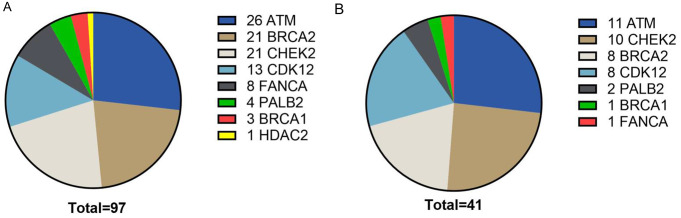
The distribution of HRR gene mutations. (**A**) Depiction of all HRR mutations found, including suspected benign variants, variants of unknown significance, suspected pathogenic and pathogenic mutations. ATM (*n* = 26) mutations were most prevalent, followed by mutation in BRCA2 (*n* = 21), CHEK2 (*n* = 21), CDK12 (*n* = 13), FANCA (*n* = 8), PALB2 (*n* = 4), BRCA1 (*n* = 3) and HDAC2 (*n* = 1). (**B**) Depiction of suspected pathogenic and pathogenic HRR mutations. ATM (*n* = 11) mutations were most prevalent, followed by mutation in CHEK2 (*n* = 10), BRCA2 (*n* = 8), CDK12 (*n* = 8), PALB2 (*n* = 2), BRCA1 (*n* = 1) and FANCA (*n* = 1). No suspected pathogenic or pathogenic mutations in HDAC2 were found

The mutations, including suspected benign variants, variants of unknown significance, suspected pathogenic or pathogenic mutations, were distributed among the cases as follows (Fig. 2A): 26 showed ATM mutations (14.2%), 21 showed BRCA2 mutations (11.5%), 21 showed CHEK2 mutations (11.5%), 13 showed CDK12 mutations (7.1%), 8 showed FANCA mutations (4.4%), 4 showed PALB2 mutations (2.2%), 3 showed BRCA1 mutations (1.6%), and 1 showed a HDAC2 mutation (0.5%). Notably, slightly less than a half of these mutations (43.6%, *n* = 48) were deemed pathogenic or suspected pathogenic according to pertinent databases, suggesting potential targetability. As anticipated, the prevalence of pathogenic or suspected pathogenic mutations was higher in metastatic tumor tissue compared to primary tumor tissue (25.0% vs. 19.4%), although this was not statistically significant (p-value 0.37). No pathogenic or suspected pathogenic mutations could be detected in tumor tissue from local recurrences (*n* = 9). Further analysis discerned that pathogenic or suspected pathogenic mutations were distributed as follows (Fig. 2B): 11 ATM mutations (6.0%), 10 CHEK2 mutations (5.5%), 8 BRCA2 mutations (4.4%), 8 CDK12 mutations (4.4%), 2 PALB2 mutations (1.1%), 1 FANCA mutation (0.6%), and 1 BRCA1 mutation (0.6%). No pathogenic or suspected pathogenic HDAC2 mutations were identified. In total, 38 out of 183 cases (20.8%) exhibited a potentially targetable mutation, while the remainder showcased variants of unknown significance (*n* = 37, 20.2%) or suspected benign variants (*n* = 4, 2.2%).

## Discussion

The positive outcomes observed in the PROfound trial have prompted a clinical imperative for testing BRCA1/2 and homologous recombination repair (HRR) gene mutations in patients with metastatic castration-resistant prostate cancer (mCRPC) [1]. The prevalence of HRR mutations in prostate cancer has been documented in numerous studies, demonstrating variations depending upon the origin of the tissue, whether primary or metastatic and the stage of disease [9–12, 14]. A large study on primary hormone-sensitive prostate cancer documented targetable HRR alterations in about 19% of cases, while the prevalence in mCRPC ranges from 19 to 33% [9–11, 14]. Herein, we present real-world data derived from the analysis of 197 prostate cancer cases over a 5-year period within a single institution.

Within our cohort, the prevalence of pathogenic or suspected pathogenic mutations was determined to be 20.8% (*n* = 38), in accordance with published data. However, in contrast to published data, the distribution of the mutations differed substantially. Our findings reveal a predominance of targetable mutations in ATM, succeeded by mutations in CHEK2 and BRCA2. Thus, targetable BRCA2 mutations were less frequently identified in our cohort. According to the literature, the prevalence of BRCA2 alterations in primary prostate cancer is significantly lower than that in mCRPC [9, 11]. The cohort presented here consists of a mixture of primary and metastatic castration-resistant tumors, which is a valid explanation for the lower prevalence of BRCA2 mutations. In concordance to the literature, BRCA1 mutations were found in less than 1% of all cases. In the context of olaparib treatment, this signifies that 20.8% (*n* = 38) meet the criteria for treatment per FDA approval, whereas only 4.9% (*n* = 9) align with the eligibility criteria for olaparib treatment according to EMA approval.

The prevalence of ATM mutations in our study is similar to that described in the literature. In mCRPC, ATM mutations are present in 5-8%, while the prevalence is two-fold lower in primary prostate cancer [9, 11, 15, 17]. ATM germline mutations are potential prognostic biomarkers because they are enriched in patients with lethal and high grade disease [18, 19]. However, this association has not been verified for somatic mutations. In addition to its role as a potential prognostic biomarker, ATM status was also described as a predictive biomarker regarding treatment response to olaparib in the initial studies [20, 21]. Yet, recent studies on PARP inhibition therapy in mCRPC indicate, that the response rate of ATM mutated tumors is lower than that of BRCA2 mutated tumors [1, 22–26].

Our data also sheds light on practical clinical management involving this novel diagnostic test. It is the metastatic tumor manifestation that drives disease progression in prostate cancer. In accordance with the published data, the prevalence of potentially treatment relevant mutations in our cohort was slightly higher in metastatic tumor tissue compared to primary tumor tissue (19.4% vs. 25.0%), although this was not statistically significant. Furthermore, primary tumor tissue exhibited greater instances of insufficient quality, likely attributable to inadequate DNA quality. Consequently, it advises to consider harvesting metastatic tumor tissue as the preferred material for optimal treatment planning. Despite this, within our cohort of 197 cases, mutation analysis was predominantly conducted on primary tumor tissue, possibly because metastatic tumor biopsy for molecular testing is still not common practice. Also, as clinicians are aware of the fact, that in nearly half of the cases HRR alterations constitute germline defects, they prefer to resort to the already existing biopsy material. However, the data of this study suggests that an additional biopsy of a bone metastasis has the potential benefit of obtaining tumor tissue with an adequate DNA quality and a slightly higher likelihood of detecting a targetable mutation.

A limitation of our study lies in the absence of data on genomic deletions, which are challenging to reliably detect using next-generation sequencing. This might also account for the comparatively lower number of alterations identified, particularly in BRCA2.

In conclusion, this unicentric analysis of BRCA1/2 and HRR gene mutations in prostate cancer, analyzed over a 5-year period, found a lower prevalence of BRCA2 mutations than previously reported: only 4.9% are deemed eligible for olaparib treatment as per EMA approval.

## References

[1] de Bono J, Mateo J, Fizazi K et al (2020) Olaparib for metastatic castration-resistant prostate Cancer. N Engl J Med 382:2091–2102. 10.1056/nejmoa1911440 32343890 10.1056/NEJMoa1911440

[2] Hussain M, Mateo J, Fizazi K et al (2020) Survival with Olaparib in Metastatic Castration-resistant prostate Cancer. N Engl J Med 383:2345–2357. 10.1056/nejmoa2022485 32955174 10.1056/NEJMoa2022485

[3] Saad F, Clarke NW, Oya M et al (2023) Olaparib plus abiraterone versus placebo plus abiraterone in metastatic castration-resistant prostate cancer (PROpel): final prespecified overall survival results of a randomised, double-blind, phase 3 trial. Lancet Oncol 24:1094–1108. 10.1016/S1470-2045(23)00382-0 37714168 10.1016/S1470-2045(23)00382-0

[4] European Medicines Agency Lynparza Product Information. https://www.ema.europa.eu/en/documents/product-information/lynparza-epar-product-information_en.pdf

[5] Fallah J, Xu J, Weinstock C et al (2024) FDA approval Summary: Olaparib in Combination with Abiraterone for treatment of patients with BRCA -Mutated metastatic castration-resistant prostate Cancer. J Clin Oncol 42:605–613. 10.1200/JCO.23.01868 38127780 10.1200/JCO.23.01868

[6] US Food and Drug Administration: FDA approves olaparib with abiraterone and prednisone (or prednisolone) for BRCA-mutated metastatic castration-resistant prostate cancer (2023) https://www.fda.gov/drugs/drug-approvals-and-databases/fda-approves-olaparib-abiraterone-and-prednisone-or-prednisolone-brca-mutated-metastatic-castration

[7] Chi KN, Sandhu S, Smith MR et al (2023) Niraparib plus abiraterone acetate with prednisone in patients with metastatic castration-resistant prostate cancer and homologous recombination repair gene alterations: second interim analysis of the randomized phase III MAGNITUDE trial. Ann Oncol 34:772–782. 10.1016/j.annonc.2023.06.009 37399894 10.1016/j.annonc.2023.06.009 PMC10849465

[8] Agarwal N, Azad AA, Carles J et al (2023) Talazoparib plus Enzalutamide in men with first-line metastatic castration-resistant prostate cancer (TALAPRO-2): a randomised, placebo-controlled, phase 3 trial. Lancet 402:291–303. 10.1016/S0140-6736(23)01055-3 37285865 10.1016/S0140-6736(23)01055-3

[9] Robinson D, Van Allen EM, Wu YM et al (2015) Integrative clinical genomics of advanced prostate cancer. Cell 161:1215–1228. 10.1016/J.CELL.2015.05.001 26000489 10.1016/j.cell.2015.05.001 PMC4484602

[10] Taylor BS, Schultz N, Hieronymus H et al (2010) Integrative genomic profiling of human prostate cancer. Cancer Cell 18:11–22. 10.1016/J.CCR.2010.05.026 20579941 10.1016/j.ccr.2010.05.026 PMC3198787

[11] Abeshouse A, Ahn J, Akbani R et al (2015) The Molecular Taxonomy of primary prostate Cancer. Cell 163:1011–1025. 10.1016/j.cell.2015.10.025 26544944 10.1016/j.cell.2015.10.025 PMC4695400

[12] Quigley DA, Dang HX, Zhao SG et al (2018) Genomic Hallmarks and Structural Variation in metastatic prostate Cancer. Cell 174:758–769e9. 10.1016/j.cell.2018.06.039 30033370 10.1016/j.cell.2018.06.039 PMC6425931

[13] Armenia J, Wankowicz SAM, Liu D et al (2018) The long tail of oncogenic drivers in prostate cancer. Nat Genet 50:645–651. 10.1038/s41588-018-0078-z 29610475 10.1038/s41588-018-0078-z PMC6107367

[14] Mateo J, Carreira S, Sandhu S et al (2015) DNA-Repair defects and Olaparib in metastatic prostate Cancer. N Engl J Med 373:1697–1708. 10.1056/nejmoa1506859 26510020 10.1056/NEJMoa1506859 PMC5228595

[15] Pritchard CC, Mateo J, Walsh MF et al (2016) Inherited DNA-Repair gene mutations in men with metastatic prostate Cancer. N Engl J Med 375:443–453. 10.1056/nejmoa1603144 27433846 10.1056/NEJMoa1603144 PMC4986616

[16] Castro E, Romero-Laorden N, Del Pozo A et al (2019) PROREPAIR-B: a prospective cohort study of the impact of germline DNA repair mutations on the outcomes of patients with metastatic castration-resistant prostate Cancer. J Clin Oncol 37:490–503. 10.1200/JCO.18.00358 30625039 10.1200/JCO.18.00358

[17] Abida W, Cyrta J, Heller G et al (2019) Genomic correlates of clinical outcome in advanced prostate cancer. Proc Natl Acad Sci U S A 116:11428–11436. 10.1073/pnas.1902651116/-/DCSupplemental 31061129 10.1073/pnas.1902651116 PMC6561293

[18] Wu Y, Yu H, Li S et al (2020) Rare germline pathogenic mutations of DNA repair genes are most strongly Associated with Grade Group 5 prostate Cancer. Eur Urol Oncol 3:224–230. 10.1016/j.euo.2019.12.003 31948886 10.1016/j.euo.2019.12.003

[19] Na R, Zheng SL, Han M et al (2017) Germline mutations in ATM and BRCA1/2 Distinguish Risk for Lethal and indolent prostate Cancer and are Associated with early age at death. Eur Urol 71:740–747. 10.1016/j.eururo.2016.11.033 27989354 10.1016/j.eururo.2016.11.033 PMC5535082

[20] Mateo J, Boysen G, Barbieri CE et al (2017) DNA repair in prostate Cancer: Biology and Clinical implications. Eur Urol 71:417–425. 10.1016/j.eururo.2016.08.037 27590317 10.1016/j.eururo.2016.08.037

[21] Mateo J, Porta N, Bianchini D et al (2020) Olaparib in patients with metastatic castration-resistant prostate cancer with DNA repair gene aberrations (TOPARP-B): a multicentre, open-label, randomised, phase 2 trial. Lancet Oncol 21:162–174. 10.1016/S1470-2045(19)30684-9 31806540 10.1016/S1470-2045(19)30684-9 PMC6941219

[22] Abida W, Campbell D, Patnaik A et al (2019) 846PD - preliminary results from the TRITON2 study of rucaparib in patients (pts) with DNA damage repair (DDR)-deficient metastatic castration-resistant prostate cancer (mCRPC): updated analyses. Ann Oncol 30:v327–v328. 10.1093/annonc/mdz248.003

[23] Smith MR, Scher HI, Sandhu S et al (2022) Niraparib in patients with metastatic castration-resistant prostate cancer and DNA repair gene defects (GALAHAD): a multicentre, open-label, phase 2 trial. Lancet Oncol 23:362–373. 10.1016/S1470-2045(21)00757-9 35131040 10.1016/S1470-2045(21)00757-9 PMC9361481

[24] Marshall CH, Sokolova AO, McNatty AL et al (2019) Differential Response to Olaparib Treatment among men with metastatic castration-resistant prostate Cancer harboring BRCA1 or BRCA2 Versus ATM mutations. Eur Urol 76:452–458. 10.1016/j.eururo.2019.02.002 30797618 10.1016/j.eururo.2019.02.002 PMC6703974

[25] De Bono JS, Mehra N, Higano CS et al (2020) TALAPRO-1: a phase II study of talazoparib (TALA) in men with DNA damage repair mutations (DDRmut) and metastatic castration-resistant prostate cancer (mCRPC)—First interim analysis (IA). J Clin Oncol 38:119. 10.1200/JCO.2020.38.6_suppl.119

[26] Luo J, Antonarakis ES (2019) PARP inhibition — not all gene mutations are created equal. Nat Rev Urol 16:4–6. 10.1038/s41585-018-0129-3 30498248 10.1038/s41585-018-0129-3 PMC7959484

